# Study Protocol of a Randomized, Two-Arm, Phase I/II Trial Investigating the Feasibility, Safety, and Efficacy of Local Treatment with US-Guided High-Intensity Focused Ultrasound in Combination with Palliative Chemotherapy in Inoperable Pancreatic Cancer

**DOI:** 10.3390/jcm13133717

**Published:** 2024-06-26

**Authors:** Milka Marinova, David-Alexis Khouri, Jim Küppers, Olga Ramig, Holger M. Strunk, Johannes Breuers, Julia Fazaal, Christine Fuhrmann, Martin Coenen, Christian Möhring, Taotao Zhou, Xin Zhou, Thomas Anhalt, Farsaneh Sadeghlar, Marcus Thudium, Rupert Conrad, Georg Feldmann, Peter Brossart, Tim R. Glowka, Jörg C. Kalff, Markus Essler, Christian P. Strassburg, Yon-Dschun Ko, Ingo G. H. Schmidt-Wolf, Maria Gonzalez-Carmona

**Affiliations:** 1 Department of Nuclear Medicine, University Hospital Bonn, 53127 Bonn, Germany; 2 Department of Diagnostic and Interventional Radiology, University Hospital Bonn, 53127 Bonn, Germany; 3 Medical Center Bonn, 53127 Bonn, Germany; 4 D Clinical Study Core Unit Bonn, Institute of Clinical Chemistry and Clinical Pharmacology, University Hospital Bonn, 53127 Bonn, Germany; 5 Department of Internal Medicine I, University Hospital Bonn, 53127 Bonn, Germany; 6 Department of Anesthesiology, University Hospital Bonn, 53127 Bonn, Germany; 7 Department of Psychosomatic Medicine and Psychotherapy, University Hospital Muenster, 48149 Muenster, Germany; 8 Department of Internal Medicine III, University Hospital Bonn, 53127 Bonn, Germany; 9 Department of Surgery, University Hospital Bonn, 53127 Bonn, Germany; 10 Oncological Center, Johanniter Hospital, 53113 Bonn, Germany; 11 Department of Integrated Oncology, CIO Bonn, University Hospital Bonn, 53127 Bonn, Germany

**Keywords:** ductal adenocarcinoma of the pancreas, high-intensity focused ultrasound, non-invasive treatment option, symptom relief, pain management

## Abstract

Background: Pancreatic adenocarcinoma (PaC) still has a dismal prognosis, and despite medical advances, a bleak 5-year survival rate of only 8%, largely due to late diagnosis and limited curative surgical options for most patients. Frontline palliative treatment shows some survival advantages. However, the high disease mortality is accompanied by high morbidity including cancer-related pain and additional symptoms, which strongly impair patients’ quality of life (QOL). At present, there is no established strategy for local therapy for PaC primarily aiming to manage local tumor growth and alleviate associated symptoms, particularly pain. In recent years, non-invasive high-intensity focused ultrasound (HIFU) has shown promising results in reducing cancer pain and tumor mass, improving patients’ QOL with few side effects. Study design: This is the first randomized controlled trial worldwide including 40 patients with inoperable pancreatic adenocarcinoma randomized into two groups: group A undergoing standard chemotherapy; and group B undergoing standard chemotherapy plus local HIFU treatment. This study aims to establish a robust evidence base by examining the feasibility, safety, and efficacy of US-guided HIFU in combination with standard palliative systemic therapy for unresectable PaC. Primary endpoint assessments will focus on parameters including safety issues (phase I), and local response rates (phase II).

## 1. Background and Rationale

### 1.1. Pancreatic Adenocarcinoma (PaC)

Pancreatic adenocarcinoma (PaC) ranks as the 5th most common cause of cancer-related deaths in the Western world [1,2,3]. Worldwide, PaC is the 8th leading cause of cancer-related death in men and 9th leading cause of cancer-related death in women, as reported by the World Health Organization (WHO). The disease incidence notably increases after the age of 45 years, with a higher occurrence in males than in females (male-to-female ratio 1.3:1). In the past decade, both the age-adjusted incidence rate and the mortality have shown a 2.1% increase [3,4,5].

Despite medical advances, PaC continues to have a dismal 5-year survival rate of only 8%. Upon diagnosis, more than 85–90% of patients with pancreatic cancer are no longer curatively operable due to locally advanced disease or presence of metastases; the median survival time in these patients is only 4–6 months and the 5-year survival rate is less than 5% [1,3,6].

To date, the most widely accepted frontline palliative treatment regimens for advanced pancreatic cancer include FOLFIRINOX (5-fluorouracil (5-FU), leucovorin (LV), irinotecan, and oxaliplatin) and the combination of gemcitabine and nanoparticle albumin-bound paclitaxel (nab-paclitaxel) [7,8]. The FOLFIRINOX regimen demonstrated a significant survival advantage of 11.1 months compared to 6.8 months for gemcitabine alone in a randomized phase III trial. The alternative frontline palliative systemic chemotherapy regimen, the combination of gemcitabine and nab-paclitaxel, yielded a survival benefit of nearly 2 months in comparison to gemcitabine alone (8.5 months vs. 6.7 months). More recently, NALIRIFOX (liposomal irinotecan, 5-FU/LV, and oxaliplatin) improved overall survival and progression-free survival compared with gemcitabine plus nab-paclitaxel in a randomized, multicentric phase III trial (NAPOLI-3-trial) [9,10]. The median overall survival was 11.1 months with NALFIRINOX versus 9.2 months with nab-paclitaxel-gemcitabine. As a second-line palliative chemotherapy, the combination of 5-FU/LV with nanoliposomal irinotecan (nal-IRI) has demonstrated effectiveness subsequent to gemcitabine-based therapy [11].

Despite the therapies available today for PaC, the combination of late diagnosis, early metastasis, and limited curative surgery significantly contribute to the high mortality rate of this disease [3]. Moreover, in over 80% of cases, the quality of life (QOL) of affected patients is substantially compromised by cancer pain, which represents the most critical clinical symptom [12]. Moreover, pain seems to be associated with a poor prognosis [13,14,15]. Additional symptoms associated with advanced primary pancreatic cancer comprise exocrine and endocrine insufficiency of the pancreas, alongside biliary obstruction and duodenal stenosis. Furthermore, other psychosocial stress factors such as fatigue, sleep disorders, and disruptions in emotional and cognitive function are present. Hence, there is an urgent need for new alternative forms of therapy, particularly in the palliative area.

### 1.2. Local Treatment Options of PaC

Local therapies for PaC aim to prevent local growth of the primary tumor, either to avoid tumor-associated local complications or to mitigate symptoms. Increasing pain symptoms represent one of the most common situations in cases of locally advanced PaC. Palliative approaches, including pharmaceutical interventions (analgesia utilizing opioids) or interventional procedures like coeliac plexus blockades, are commonly employed to address the pain syndrome [16,17]. However, opioid therapy is often associated with stressful adverse effects, including obstipation, and respiratory depression. The celiac plexus block, while being a less invasive approach, generally offers only transient and short-term relief of the pain symptoms [18,19].

At present, there is no definitive strategy for the local treatment of PaC. While radiotherapy currently stands as the most established local treatment method, additional local ablation methods have been employed with varying degrees of success in recent years. These local treatment options include radiofrequency ablation, microwave ablation, irreversible electroporation, and high-intensity focused ultrasound (HIFU) [20,21,22]. These approaches facilitate the induction of coagulation necrosis directly within the tumor tissue through the generated energy. However, with the exception of HIFU, most procedures, using either a surgical or transcutaneous approach, range from minimally invasive to invasive as they require the insertion of needles, probes, or electrodes into the target tissue to generate local energy and achieve their function.

### 1.3. High-Intensity Focused Ultrasound (HIFU)

HIFU is an innovative procedure that enables targeted, non-invasive thermal ablation of tissue accessible to sonography. In contrast to diagnostic ultrasound (US), HIFU generates much higher energies with temperatures rising up to 80 °C and causes coagulation necrosis in the target tissue [23]. The ultrasonic waves are bundled by special transducers and can thus be focused on tissue measuring a few millimeters within the target region. Magnetic resonance imaging (MRI) and sonography are available for locating the tumor and providing imaging control of the procedure. The use of HIFU has been previously reported for a variety of diseases, e.g., uterine fibroids, peripheral bone tumors, liver tumors and pancreatic tumors [24,25,26,27].

At present, US-guided HIFU is available as a treatment option for PaC in specialized centers worldwide. Although the procedure is not yet standardized, HIFU has proven to be a safe and effective treatment for advanced PaC in palliative settings, leading to significant reductions in cancer pain and tumor mass. Consequently, it enhances the QOL and physical comfort of patients with a very low rate of side effects [26,28,29,30,31,32,33,34]. HIFU operates without the need for needles, electrodes, or probes, making it a viable treatment option even for patients with tumors located in close proximity to blood vessels, bowel loops, or biliary stents. Further, potential complications associated with punctures, such as bleeding, or the risk of seeding metastases along the puncture channel, are not a concern with HIFU. Its non-invasive nature serves as a significant advantage, positioning HIFU as a valuable contributor alongside supportive care in the comprehensive management of this challenging disease.

Tumor ablation using HIFU is generally considered a low-risk, non-invasive procedure, with few and generally mild side effects. Our previous experience and results confirm this, even for tumors encasing important vessels in the upper abdomen. Side effects are rare and include transient discomfort or pain, skin changes, mild fever, rare instances of injury to adjacent organs or vessels, infection, and pancreatic enzyme increase.

### 1.4. Rationale of the Study

At present, results from prospective randomized controlled trials investigating the potential of HIFU in achieving local tumor control and clinically significant improvement in patients with unresectable PaC are not available. While HIFU technology complements existing interventional techniques in oncology, its non-invasiveness and comparatively low-risk nature justifies its consideration as a symptomatic treatment for locally advanced PaC. However, further evidence is necessary to validate the role of HIFU as a local therapy for this disease.

Therefore, based on the above findings, we aim to contribute to the establishment of a solid evidence base by investigating the feasibility, safety and efficacy of local therapy with US-guided HIFU in combination with standard palliative systemic therapy in patients with unresectable pancreatic cancer in a prospective randomized controlled two-arm phase I/II study. Through the assessment of parameters such as tumor volume, symptoms, and QOL using standardized evaluation methods (RECIST 1.1, standardized questionnaires), the effects of local HIFU ablation on local tumor control and symptom management, e.g., pain relief, will be under evaluation. Moreover, the impact on progression-free survival and overall survival will be evaluated.

Ultimately, the insights obtained from this study may aid in identifying patients who would benefit most from the combination of HIFU therapy and standard chemotherapy. In addition, the generation of evidence-based data is crucial for the development of larger, multicenter, randomized controlled trials. Moreover, ongoing assessments are examining the potential activation of the immune system using HIFU.

## 2. Trial Methodology

### 2.1. Trial Design

**Figure 1:**
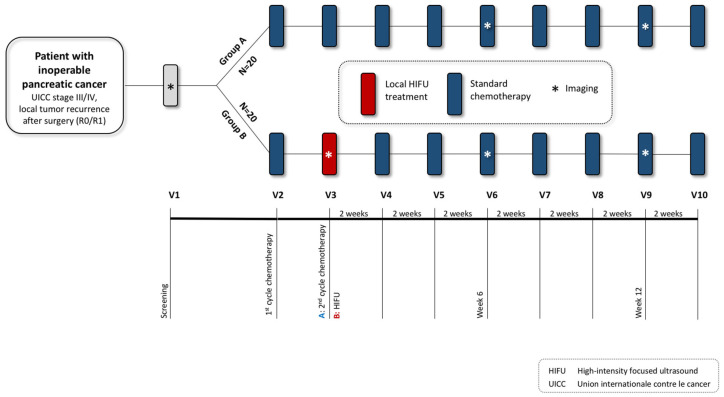
Intervention scheme: Control intervention (Group A): standard palliative chemotherapy. Experimental intervention (Group B): additional local HIFU ablation to standard palliative chemotherapy. Duration of intervention per patient: 4 months. Time of data collection: at baseline, every visit 1–10, every 3 months till death.

**Figure 2:**
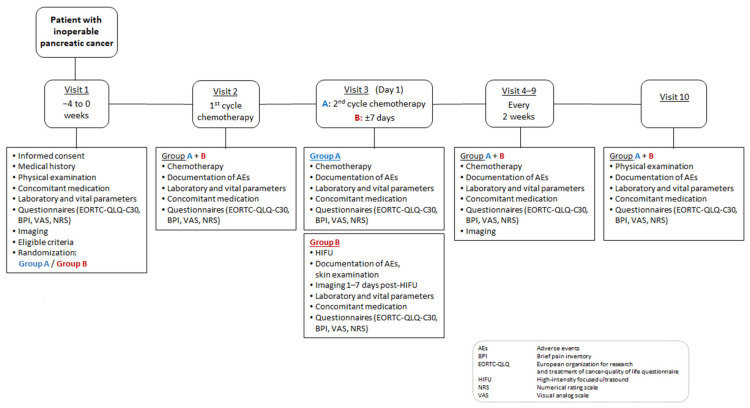
Trial flow.

This is a prospective, monocentric, randomized, two-arm, open-label clinical trial (Figure 1). Forty patients with inoperable pancreatic cancer (locally advanced pancreatic cancer, primary metastatic pancreatic cancer, local recurrence after R0 or R1 resection of pancreatic cancer, UICC stage III-IV) will be randomized into two groups: group A undergoing standard chemotherapy; and group B undergoing standard chemotherapy plus local HIFU (after the first cycle of systemic chemotherapy). The HIFU procedure typically lasts for 1–4 h, with a hospital stay of 2–3 days. Each patient will be monitored for a follow-up period of 4 months as part of the scheduled visits (Figure 1 and Figure 2, Table 1). Data will then be collected every 3 months until death or failure to follow-up. The clinical trial will be conducted in the interdisciplinary HIFU center at the University Hospital Bonn, and all investigators meet the requirements to perform the planned study-specific examinations and therapies.

### 2.2. Study Population

Forty patients with cytologically or histologically confirmed, non-operable adenocarcinoma of the pancreas and patients with indication for first-line palliative chemotherapy (gemcitabine/nab-paclitaxel; FOLFIRINOX; NALIRIFOX) or second-line chemotherapy (gemcitabine-based chemotherapy; 5-FU/LV plus Nal-IRI (liposomal irinotecan)) will be recruited for this study. Potential participants presenting at the gastroenterology and oncology departments of the University Hospital Bonn and the Johanniter Hospital Bonn will be informed about the study and given sufficient time to reflect before providing their written informed consent to participate. The selection criteria for participation in the clinical trial are presented in Table 2.

Patients will then be randomized into two groups: group A will receive standard chemotherapy, and group B will receive standard chemotherapy in combination with local HIFU treatment. Stratification will be based on the stage of disease (locally advanced PaC in UICC stage III or PaC with distant metastases in UICC stage IV) and type of chemotherapy (first-line or second-line).

### 2.3. Primary Objective and Primary Endpoints

The primary objective of this randomized controlled trial (RCT) is to evaluate the safety and tolerability of local HIFU treatment in combination with standard chemotherapy compared to standard chemotherapy only in patients with advanced PaC (phase I). This involves evaluating the type, frequency, and severity of treatment-associated adverse events (AEs). AEs will be graded according to the Common Terminology Criteria for Adverse Events (CTCAE, Version 5.0), with grades 1–5 indicating increasing severity.

In addition, the expected procedure-related AEs will be recorded, including pain immediately after the HIFU ablation (measured on a pain scale NRS/VAS 0–10, with 0 indicating “no pain” and 10 indicating “the most severe pain one can imagine”), pain within the first day post-intervention, skin edema, skin redness, skin burning, and ascites.

In addition, tumor volume will be assessed using imaging (magnetic resonance tomography (MRI), and computed tomography (CT)) (baseline volume compared to tumor volume at 1 week, 6 weeks, 3 months follow-up, and every 3 months thereafter until failure to follow-up or death) to evaluate local tumor response rates (phase II).

### 2.4. Secondary Endpoints

The following objectives are defined as secondary endpoints:

- Evaluation of pain severity as the leading symptom using NRS/VAS (measured on a pain scale 0–10, with 0 indicating “no pain” and 10 indicating “the most severe pain one can imagine”), BPI questionnaire, and records on analgesic medication (at baseline, 1 week, 6 weeks, 3 months, and every 3 months thereafter till failure to follow-up or death).
- Evaluation of tumor-related symptoms and QOL using the EORTC-QLQ-C30 questionnaire (baseline scores versus scores at 1 week, 6 weeks, 3 months follow-up, and every 3 months thereafter till failure to follow-up or death).
- Evaluation of the correlation between parameters of HIFU intervention (sonication time, treatment time, total energy, and energy per milliliter treated tumor volume), achieved non-perfused volume (NPV), and tumor shrinkage over time.
- Effects of local HIFU treatment on blood parameters.
- Comparative assessment of progression-free survival, and overall survival.
- Exploratory analysis including immune parameters in peripheral blood at baseline and post-HIFU: assessments of changes in circulating immune cell populations (T cells, B cells, NK cells, and regulating T cells), cytokine levels, and immune checkpoint molecules.
- Correlation of changes in immune parameters with clinical outcomes like tumor size reduction.
- Exploration of potential biomarkers that could predict responses to HIFU, including novel cytokines, immune cells, or gene expression patterns.

## 3. Special Aspects

### 3.1. Statistical Analysis

The data obtained from this RCT will be analyzed using SPSS (version 27 SPSS Inc., Chicago, IL, USA) and Stata (version 17, StataCorp, StataCorp LP, College Station, TX, USA). Means and their confidence intervals, medians, standard deviations, and ranges will be determined. Statistical evaluation of tumor volumes, pain scores (according to NRS/VAS, BPI), QOL scales (EORTC-QLQ-C30, version 3.0, in its validated German version), and blood parameters will be performed using longitudinal mixed models evaluating baseline values (before start of palliative therapy) and corresponding values at different time points (visit 1–10 within the first 4 months, and every 3 months thereafter till failure to follow-up or death). Other possible influential factors (principle and raised confounders) including age, stage of disease, sex, and chemotherapy regimen, will be tested using mixed-models. Mixed-effects models for repeated measures will be applied to the longitudinal data using a random effect to account for the correlation between repeated outcome measurements on the same individual. A *p*-value of <0.05 is considered statistically significant.

### 3.2. Risks and Complications Regarding Standard Chemotherapy

It is expected that this study will not result in any other adverse effects beyond the known side effect profile of conventional polychemotherapy. Expected side effects of systemic chemotherapy for PaC patients may include but are not limited to fatigue, nausea, vomiting, loss of appetite, and hair loss. Additionally, patients might experience lowered blood cell counts, mostly neutropenia, resulting in an increased risk of infections, bleeding problems, or fatigue. Gastrointestinal symptoms such as diarrhea or constipation, and mucositis, as well as neuropathy leading to tingling or numbness in the extremities, are also common. It is important to note that the severity of these side effects can vary among individuals and may require close monitoring and supportive care to manage and alleviate their impact.

### 3.3. Risks and Complications Regarding Local HIFU Treatment

Based on current knowledge, US-guided HIFU treatment for advanced PaC is a low-risk procedure that is generally associated with a very low rate of adverse effects and procedure-associated complications, taking into account the indications and contraindications [35]. All contraindications to the procedure will be asked in detail during the informed consent process, and if contraindications are present, the patient will not be included in the study.

Probable procedure-related adverse effects to the patient include:

- Mild discomfort or pain in the upper abdomen and/or on the skin overlying the treated tumor (usually of a short duration, up to 12 h after the intervention)
- Skin changes (in <5% of patients): mild redness, cutaneous edema, skin burning, and induration of subcutaneous adipose tissue
- Mild fever (in about 5–10% of cases; for up to 48 h post-procedure)
- Mild inflammatory reaction with blood count changes and CRP rises
- Injury to adjacent organs and vessels (very rare, in <1% of cases)
- Infection of the necrosis cavity with consecutive need for surgery or puncture
- Increase in pancreatic enzymes with or without signs of clinical pancreatitis (1.9%)
- Other very rare possible risks and complications are related to bleeding (0.1%), worsening of pre-existing jaundice (0.6%), occlusion of tumor-involved vessels, e.g., superior mesenteric artery (0.06%), steatorrhea (0.8%), gastrointestinal dysfunction (0.8%), positional damage (0.1%), peritonitis, pancreatic pseudocyst formation, and intestinal perforation (<0.01%).

### 3.4. Ethical Considerations and Trial Registration

This study has been initiated after professional ethical consultation by the responsible ethics committee at the University Hospital Bonn, University of Bonn (No. 307/15, last amendment January 2024). The clinical trial is being conducted in accordance with the study protocol and the regulations of the Declaration of Helsinki (Edinburgh, Scotland, October 2000), the Good Clinical Practice (GCP) Regulation and the ICH (International Council for Harmonisation). A written informed consent will be obtained from all participants. This study is registered at the German Clinical Trials Register (Deutsches Register Klinischer Studien, DRKS00012367).

## 4. Conclusions

Previous clinical data have demonstrated that non-invasive, US-guided HIFU is an effective and low-risk treatment option for advanced PaC, as long as the tumors are accessible to therapeutic US. HIFU provides effective local tumor and symptom control alongside standard palliative care. This RCT will evaluate the safety, efficacy, and clinical outcomes of US-guided HIFU treatment in 40 patients with advanced PaC, randomized into two groups: group A with standard palliative chemotherapy, and group B with additional local HIFU ablation to standard palliative chemotherapy. Clinical outcomes will be comprehensively and comparatively evaluated concerning local tumor growth, the progression of symptoms and QOL during the disease course, particularly emphasizing the primary symptom of cancer pain, and survival (overall survival and progression-free survival). The aim of the RCT is to identify the patients who would benefit most from this additional innovative local treatment and to gather evidence-based data for future research in a broader, multicenter approach.

## Figures and Tables

**Table 1:** Schedule of activities.

		Visit 1	Visit 2	Visit 3		Visit 4	Visit 5	Visit 6	Visit 7	Visit 8	Visit 9	Visit 10
		−4 to 0 weeks	1st cycle chemo- therapy	Day 1/ 2nd cycle chemo- therapy	Day 1/ 2nd cycle (±7 days) HIFU	+2 weeks/ chemo- therapy	+2 weeks/ chemo- therapy	+2 weeks/ chemo- therapy	+2 weeks/ chemo- therapy	+2 weeks/ chemo- therapy	+2 weeks/ chemo- therapy	Final visit
				A	B							
Screening	Written informed consent	x										
	Medical history	x										
	Physical examination	x										x
	Eligibility criteria	x										
Randomization		x										
Laboratory parameters	Tumor marker (CEA, CA 19-9, Cyfra 21-1)	x	x	x	x	x	x	x	x	x	x	x
	Blood count	x	x	x	x	x	x	x	x	x	x	x
	Infection parameters	x	x	x	x	x	x	x	x	x	x	x
	Coagulation parameters	x	x	x	x	x	x	x	x	x	x	x
	Liver enzymes	x	x	x	x	x	x	x	x	x	x	x
	Pancreas enzymes	x	x	x	x	x	x	x	x	x	x	x
	Creatinine/GFR	x	x	x	x	x	x	x	x	x	x	x
	Immune status	x	x	x	x	x	x	x	x	x	x	x
	ß-HCG	x										
Vital signs	Blood pressure, heart rate, temperature, weight, height	x	x	x	x	x	x	x	x	x	x	x
Imaging	MRI, CT	x			x			x			x	
	US	x			x			x			x	
Documentation of AEs	Documentation of AEs	x	x	x	x	x	x	x	x	x	x	x
Drug history	Concomitant medication	x	x	x	x	x	x	x	x	x	x	x
ECOG status documentation		x	x	x	x	x	x	x	x	x	x	x
Questionnaires	EORTC-QLQ-C30	x	x	x	x	x	x	x	x	x	x	x
	VAS, NRS, BPI	x	x	x	x	x	x	x	x	x	x	x
Chemotherapy	Group A, B		x	x		x	x	x	x	x	x	x
HIFU (Group B only)					x							
Analgesic medication		x	x	x	x	x	x	x	x	x	x	x

AEs = Adverse events; ß-HCG = Beta-human chorionic gonadotropin; BPI = Brief pain inventory; CA 19-9 = Carbohydrate antigen 19-9; CEA = Carcinoembryonic antigen; CT = Computed tomography; Cyfra 21-1 = Cytokeratin-19 fragment; ECOG = Eastern cooperative oncology group; EORTC-QLQ = “European organization for research and treatment of cancer-quality of life questionnaire”; GFR = Glomerular filtration rate; HIFU = High-intensity focused ultrasound; MRI = Magnetic resonance imaging; NRS = Numerical rating scale; US = Ultrasound; VAS = Visual analog scale.

**Table 2:** Selection criteria for study participation.

Inclusion Criteria	Exclusion Criteria
Age ≥ 18 years Written informed consent Capability to follow study instructions and adhere to the necessary study visits ECOG ≤ 2 Adenocarcinoma of the pancreas Histologically/cytologically confirmed Inoperable, locally advanced tumor with a diameter ≥ 2 cm Indication for first-line palliative chemotherapy or current or second-line chemotherapy Tumor sufficiently visualized with ultrasound Safe acoustic access to the tumor on ultrasound Distance between skin surface and deepest focus in the tumor max. 11 cm Adequate organ function: Blood count: absolute neutrophiles ≥ 1.5 × 10^9^/L; hemoglobin ≥ 8 g/dL; platelet count ≥ 75 × 10^9^/L Renal function: normal range creatinine or creatinine clearance ≥ 50 mL/min (Cockcroft and Gault). Liver function: AST/ALT ≤ 5× of normal; bilirubin < 4 mg/dL Expected survival of more than 3 months Eligibility for general anesthesia Excluded pregnancy (negative pregnancy test, ß-HCG)	Participation in another clinical trial with intake of an investigational drug up to 30 days prior to participation in this clinical trial Previous intervention: - Exploratory laparotomy less than 6 weeks ago - Local therapies for pancreatic cancer (e.g., radiotherapy, cryotherapy) Extensive scarring along the acoustic pathway Open wounds or unhealed scars after previous surgery of the anterior wall in the acoustic pathway Acute serious infection (e.g., cholangitis) Multiple metal clip materials in the upper abdomen after surgery Bowel infiltration with bleeding
